# The Interspinous Spacer: A Clinicoanatomical Investigation Using Plastination

**DOI:** 10.1155/2012/538697

**Published:** 2012-08-01

**Authors:** Thomas Kaulhausen, Kourosh Zarghooni, Gregor Stein, Jutta Knifka, Peer Eysel, Juergen Koebke, Rolf Sobottke

**Affiliations:** ^1^Department of Orthopaedic and Trauma Surgery, University Hospital of Cologne, Joseph-Stelzmann-Strabße. 9, 50931 Cologne, Germany; ^2^Center of Anatomy, Cologne University, Joseph-Stelzmann-Straße 9, 50931 Cologne, Germany

## Abstract

*Purpose*. The relatively new and less-invasive therapeutic alternative “interspinous process decompression device (IPD)” is expected to result in improved symptoms of neurogenic intermittent claudication (NIC) caused by lumbar spinal stenosis. The aim of the study was to analyze IPD position particularly regarding damage originating from surgical implantation. *Methods*. Anatomic assessments were performed on a fresh human cadaver. For the anatomic examination, the lumbar spine was plastinated after implantation of the IPDs. After radiographic control, serial 4 mm thick sections of the block plastinate were cut in the sagittal (L1–L3) and horizontal (L3–L5) planes. The macroanatomical positioning of the implants was then analyzed. The insertion procedure caused only little injury to osteoligamentous or muscular structures. The supraspinous ligament was completely intact, and the interspinous ligaments were not torn as was initially presupposed. No osseous changes at the spinal processes were apparent. Contact of the IPD with the spinous processes was visible, so that sufficient biomechanical limitation of the spinal extension seems likely. *Conclusions*. Minimally invasive IPD implantation with accurate positioning in the anterior portion of the interspinous place is possible without severe surgical trauma.

## 1. Introduction

Lumbar spinal stenosis (LSS) with neurogenic intermittent claudication (NIC) is one of the most common degenerative spinal diseases in the elderly [1–3].NIC is a specific symptom complex occurring in patients with LSS.

NIC is characterized by increasing leg, buttock, and/or groin pain with or without lower back pain when walking a certain distance or reclining. Forward bending or sitting leads to rapid pain relief.

LSS is seen frequently in clinical practice. 3 to 4% of all patients consulting a general physician with pain in the lower back region have LSS. Nearly 15% of the patients who see a specialist for lower back pain have LSS [4]. Annual incidence rates of 5/100,000 have been reported [5]. In the United States, the cost of NIC to society from medical treatment and loss of productive work hours reaches tens of billions of dollars annually [6].

Nonoperative therapy is initially considered with oral nonsteroidal anti-inflammatory drugs (NSAIDs), other analgetics, and physical therapy. This regimen can be intensified by adding epidural pain treatment (steroids, opioids, and local anesthetics). In a third of all cases, this therapy decreases symptoms sufficiently that operative treatment can be avoided. In the remaining two-thirds, surgical intervention is necessary [7].

For LSS patients over 65 years undergoing surgery, open decompression is most frequently performed [1, 8, 9]. One problem associated with decompression procedures is trauma to the osteoligamentous structures, which vares in severity depending on the extent of surgery performed. 

A relatively new and less invasive therapeutic alternative is insertion of an interspinous process decompression device (IPD). These implants are inserted between the spinal processes and are expected to result in improved symptoms. The use of interspinous implants has grown markedly over the past few years. 

Biomechanical studies have shown that IPDs significantly reduce intradiscal pressure as well as facet load, and they prevent narrowing of the spinal canal and neural foramina [10, 11]. Previous studies have shown benefits with the use of implanted devices (e.g., X-Stop) versus conservative therapy, especially with regards to the quality of life [6, 12]. 

For some patients with LSS, IPDs may be a viable alternative to open decompression [13]. IPDs may be used either as “stand alone” implants or to augment open decompression by preventing instability [14]. The main principle behind their design is the limitation of dynamic extension in the affected segment [13]. Radiologic studies have demonstrated that the use of interspinous devices affects spinal alignment as well as the dimensions of the spinal canal and neural foramina [15–17].

In addition, insertion of an IPD can be accomplished percutaneously through a 1.5 cm incision. This method is used for implantation of the Aperius PercLID device designed by Medtronic, Inc. This device has been on the market since 2006 and is CE certified. The inner core and outer shell of the implant are made of titanium (Ti-6Al-4V) with unfoldable fins. The Aperius PercLID is suitable for patients with degenerative lumbar spinal stenosis and can be implanted at the levels L1–L5. The typical candidate for this particular IPD is over 50 years of age with mild-to-moderate LSS symptoms (e.g., increasing leg, buttock, and/or groin pain with or without lower back pain when walking a certain distance or reclining), in whom conservative therapy has failed to bring sufficient relief. Most importantly, candidates for placement must report about an improvement of NIC by lumbar flexion and have undergone at least 6 months of failed nonsurgical treatment.

A number of studies published recently have shown significant clinical improvement after insertion of the Aperius PercLID implant [18–21].

One point of discussion is the relevance of damage to the posterior soft-tissue structures after implant insertion, although this depends highly on the choice of implant [22, 23].

To date, no clinicoanatomical investigations of interspinous spacers for the lumbar spine using sheet plastinates are available in the literature. The aim of the study is to evaluate macroscopic findings after IPD implantation by using the plastination techniques.

## 2. Materials and Methods

Four interspinous “stand alone” spacers (14 mm Aperius PercLID; Medtronic, Tolochenaz, Switzerland) were percutaneously implanted into the lumbar spine (L1–L5) of a fresh human cadaver, after which the segment specimens underwent plastination. The age of the female human cadaver was 83 years, and the lumbar spine had undergone no prior surgery. 

For implantation, the body was placed in a prone position. After identification of the L4/5 segment by fluoroscopy, the skin incision (length 1.5 cm) was made 10 cm lateral to the midline. The 8 mm trocar was first introduced and placed in the anterior part of the interspinous space, guided by fluoroscopy. The 8 mm trocar was then removed and replaced by the 10 mm trocar. This procedure was repeated with the 12 mm and 14 mm trocars until sufficient distraction of the spinous processes was attained. The 14 mm IPD was then implanted. Device insertion to the interspinous space was guided by fluoroscopy. The fins of the implant were then unfolded and the insertion instrument disconnected. IPD implantation to the remaining lumbar segments proceeded in some fashion. The surgical procedure was the same which would be used in a patient. 

After completion of the surgical procedures and isolation of the lumbar spine, fixation with 4% formaldehyde solution, careful dehydration and degreasing, and forced impregnation with epoxy resin (Biodur E12, Biodur E6, Biodur E600, BIODUR Products, Heidelberg, Germany) procedures were performed to attain block plastination [24]. 

Dehydration and degreasing with acetone were conducted until the water content was <0.5%. The solution was changed every four weeks. Due to the size of the sample, this process lasted 12 months. After radiographic control, serial 4 mm thick sections of the block plastinate were cut using a precision diamond-blade saw (Well Diamantdrahtsägen GmbH, Mannheim, Germany) in the sagittal (L1–L3) and horizontal (L3–L5) planes. 

To increase transparency of the obtained cuts, secondary sheet plastination (Biodur E12, Biodur E1, Biodur AE 30, BIODUR Products, Heidelberg, Germany) was performed in a flat chamber. 

After completion of plastination, the osteoligamentous structures and macroanatomical positioning of the implants were optically analyzed.

## 3. Results 

**Figure 1:**
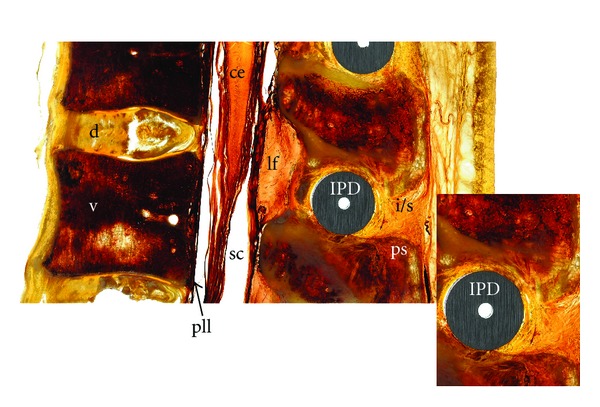
Sagittal cut with enlargement of the interspinous ligament. ce: conus medullaris; d: disc; f: intervertebral foramen; fj: facet joint; IPD: interspinous process device; i/s: inter/supraspinous ligament complex; lf: ligamentum flavum; mi: iliocostalis muscle; ml: longissimus thoracis muscle; mm: multifidus muscle; mp: psoas muscle; ms: spinalis muscle; nr: nerve root; pll: posterior longitudinal ligament; ps: spinous process; pt: transverse process; sc: spinal canal; tlf: thoracolumbar fascia; v: vertebra.

25 sagittal cuts and 25 cross-sectional cuts were obtained. The inferior and superior spinous processes showed no fracture and remained completely identifiable in the sagittal plane. The implant was positioned within the anterior part of the interspinous space. The distance of the IPD to the inferior and superior layer of the spinous processes was minimal. Osseous contact with the processes appeared in all sheets (Figure 1). 

**Figure 2:**
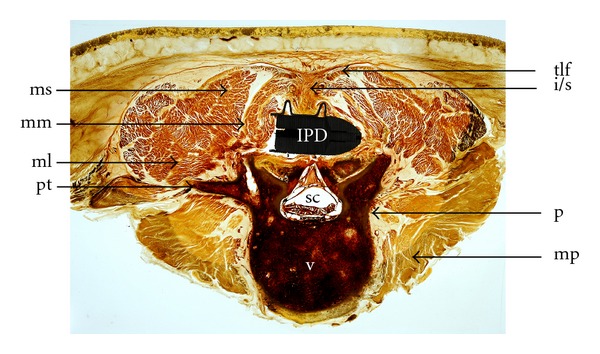
Horizontal cut segment L4/5.

In the sagittal plane both the superior and inferior spinous processes were mostly apparent, the anterior 2/3 of the interspinous ligament (ISL) was not discernible with the IPD in place. The visualized posterior 1/3 was undamaged. Complete integrity of the supraspinous ligament (SSL) was maintained (Figure 1). Furthermore, the thoracolumbar fascia and paraspinous musculature bordering the ISL/SSL, in particular the multifidus muscle, remained undamaged on sagittal and axial plane cuts (Figure 2). 

**Figure 3:**
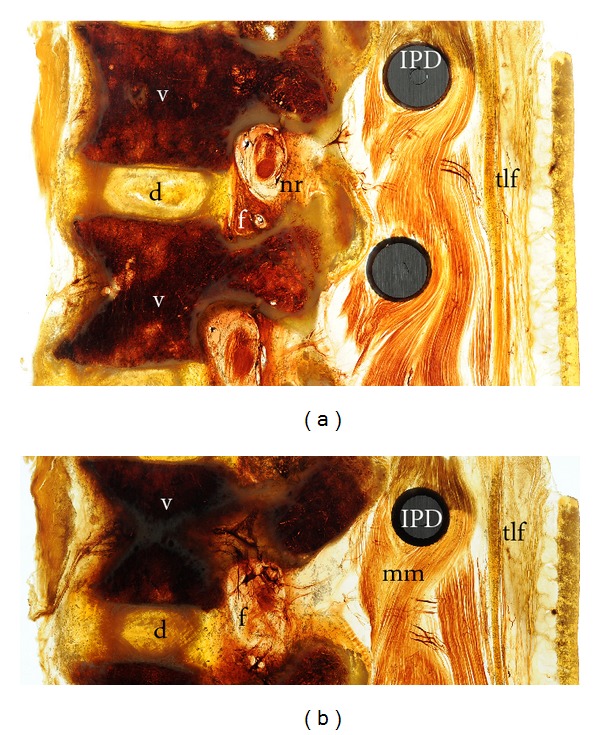
Paramedian sagittal cut with exposure of the intervertebral foramen and the normally placed nerve root.

The nerve roots were well delineated within the vertebral foramina. The spinal canal with the cauda equina and the filum were evident. Structures surrounding the spinal canal like the ligamentum flavum, the discal space, and the vertebral bodies were not distorted by the implant. The annulus fibrosus and the nucleus pulposus were clearly visible between the vertebral bodies of the segment (Figures 1, 3(a), and 3(b)). The psoas muscle formed the anterior border of the segment and was normal (Figure 2). 

## 4. Discussion

LSS is caused by degenerative changes within the spinal canal, for example osseous or ligamentous hypertrophy, disc protrusion, and/or degeneration of the intervertebral disc with instability [25]. One minimally invasive treatment option that improves patient complaints is the implantation of an interspinous spacer. 

Various studies have found that IPD placement in patients with degenerative LSS decreased symptoms [6, 12, 18–21, 26]. 

Previous studies have focused on the biomechanical effectiveness of the IPDs [10, 11].

The standard posterior midline approach to the spine has been associated with significant muscle morbidity,including muscle denervation, increased intramuscular pressure, ischemia, revascularization injury, and ligamentous damages [27–30].In an effort to minimize this type of morbidity associated with open spine procedures, recent advances in minimal access technologies have led to implementation of minimally invasive approaches to all regions of the spine for decompression, arthrodesis, and instrumentation. 

However, because IPDs are placed between the spinal processes, varying degrees of damage to the interspinous and supraspinous (ISL/SSL) ligaments are still possible. 

The structures posterior to the lumbar spine are important for supporting the spine and preventing instability. For instance, the synergy of the ISL and SSL plays an important role in stability and limiting flexion [31, 32]. A biomechanical investigation concluded that the interconnections between the supraspinous and interspinous ligaments account for as much flexion stability as each of the supraspinous and interspinous ligaments [32]. The intricate collagen fiber cross-linking between the ISL, SSL, and thoracolumbar fascia, as well as the fixation to the spinous process, lend stability and extension to the lumbar spine during abdominal muscle contraction [33]. In any case, along with the paraspinous musculature, the ISL/SSL complex plays a significant role in the stabilization of the respective vertebral segments, and not only through limitation of flexion [9, 22, 32, 34]. 

Not only the direct injury of the posterior ligament structure but also the magnitude of approach-induced changes and degeneration of these structures particularly the ISL/SSL complex, are problematic. In an animal study, the Wiltse approach led to degeneration and therefore significant biomechanical weakening of the ISL/SSL without causing direct lesions, presumably from scar formation and muscle spasms [35]. More marked degeneration, also of the neighboring vertebral segments, occurred after more invasive stabilizing and destabilizing (e.g., facetectomy) procedures [35]. 

Thus, to protect the integrity of the posterior structures and their functions as stabilizer and proprioceptive intermediaries, the most minimally invasive technique available should be selected.

To our knowledge, this study is the first to use plastination techniques to evaluate macroscopic findings after IPD implantation.

The aim of the study was to analyze IPD position particularly regarding damage originating from surgical implantation. The insertion procedure caused no injury to osteoligamentous or muscular structures. The supraspinous ligament was completely intact and the interspinous ligaments were not torn as was initially presupposed; they were merely displaced by the implant in the anterior 2/3.

No osseous changes at the spinal processes were apparent. Contact of the IPD with the spinous processes was adequate, so that sufficient biomechanical limitation of the spinal extension seems likely. 

## 5. Conclusion

 Minimally invasive IPD implantation with accurate positioning in the anterior portion of the interspinous place is possible without severe surgical trauma.

Forced impregnation with epoxy resin and subsequent secondary sheet plastination is an excellent technique for examining spine implants regarding anatomical relationships.

An analysis under dynamic loading, flexion, or extension of the vertebral column is not possible with this technique; however, any microradiographic investigations are conceivable.
